# Association of BTRC rs10883617 with Survival Outcomes in Surgically Resected Non-Small Cell Lung Cancer

**DOI:** 10.3390/genes17101166

**Published:** 2026-09-22

**Authors:** Seongwon Shin, Chi Young Jung, Mi Jeong Hong, Jang Hyuck Lee, Won Kee Lee, Ji Eun Park, Yong Hoon Lee, Hyewon Seo, Jaehee Lee, Shin Yup Lee, Sun Ha Choi, Seung Ick Cha, Chang Ho Kim, Young Woo Do, Eung Bae Lee, Seung Soo Yoo, Jin Eun Choi

**Affiliations:** 1 Lung Cancer Center, Kyungpook National University Chilgok Hospital, Daegu 41404, Republic of Koreashinyup@knu.ac.kr (S.Y.L.);; 2 Department of Internal Medicine, Daegu Catholic University School of Medicine, Daegu 41944, Republic of Korea; 3 Cell and Matrix Research Institute, School of Medicine, Kyungpook National University, Daegu 41944, Republic of Korea; 4 Biostatistics, Medical Research Collaboration Center in Kyungpook National University Hospital, School of Medicine, Kyungpook National University, Daegu 41944, Republic of Korea; 5 Department of Internal Medicine, School of Medicine, Kyungpook National University, Daegu 41944, Republic of Korea; 6 Department of Thoracic Surgery, School of Medicine, Kyungpook National University, Daegu 41944, Republic of Korea; ywd@knu.ac.kr (Y.W.D.);

**Keywords:** BTRC, rs10883617, non-small cell lung cancer, prognosis, E3 ubiquitin ligase, promoter activity

## Abstract

Background/Objectives: Genetic variation in components of the ubiquitin–proteasome system may contribute to cancer progression and clinical outcomes. This study investigated whether potentially functional variants in E3 ubiquitin ligase-related genes are associated with survival outcomes after surgical resection of non-small cell lung cancer (NSCLC). Methods: The study included 744 Korean patients with stage I–IIIA NSCLC, divided into discovery and validation cohorts. Fifty-one potentially functional single-nucleotide polymorphisms in E3 ubiquitin ligase-related genes were evaluated for associations with overall survival (OS) and disease-free survival (DFS). Results: Among the polymorphisms examined, BTRC rs10883617 T>C was significantly associated with DFS in the validation cohort, whereas the association with OS showed a concordant trend but did not reach statistical significance. In the combined cohort, carriers of at least one C allele had significantly better OS (HR = 0.55, 95% CI = 0.34–0.90, *p* = 0.02) and DFS (HR = 0.64, 95% CI = 0.46–0.89, *p* = 0.01) than patients with the TT genotype. The C allele demonstrated significantly lower promoter activity than the T allele in A549 and H1299 lung cancer cell lines (both *p* < 0.01). BTRC mRNA expression was significantly higher in tumor tissues than in paired non-neoplastic tissues (*p* < 0.001), but did not differ by genotype. Conclusions: Collectively, BTRC rs10883617 T>C was associated with favorable survival outcomes and allele-specific promoter activity in surgically resected NSCLC. However, its effect on endogenous BTRC expression remains unconfirmed in this patient population. Independent external validation and further mechanistic studies are warranted.

## 1. Introduction

Lung cancer is the most commonly diagnosed malignancy and remains the leading cause of cancer-related death worldwide [1]. Although surgical resection offers the greatest potential for long-term survival in patients with early-stage non-small cell lung cancer (NSCLC), outcomes vary considerably even among patients with comparable pathological stages. This variability suggests that tumor behavior and prognosis are determined not only by clinicopathologic characteristics but also by inherited genetic variation that may influence cancer progression and host–tumor interactions [2].

Single-nucleotide polymorphisms (SNPs) are the most prevalent form of genomic variation and are increasingly recognized as contributors to interindividual differences in cancer susceptibility, progression, and survival. In particular, potentially functional SNPs located within regulatory regions, including promoters, untranslated regions, and enhancer elements, may alter transcriptional activity and gene expression without changing protein-coding sequences [3]. These variants may therefore exert subtle but biologically relevant effects on cellular pathways involved in tumor growth, stress responses, and disease progression.

The ubiquitin–proteasome system is a fundamental regulatory pathway that maintains protein homeostasis through selective ubiquitination and proteasomal degradation, thereby governing essential cellular processes, including cell-cycle control, apoptosis, DNA damage responses, and signal transduction [4,5]. E3 ubiquitin ligases are central components of this system because they confer substrate specificity through degron recognition and regulate substrate ubiquitination and protein stability [6,7]. Dysregulation of ubiquitin-mediated protein turnover can promote cancer progression through the aberrant stabilization or degradation of key regulatory proteins [8]. Moreover, disruption of this pathway has been associated with adverse clinical outcomes, including tumor recurrence and reduced survival [9,10].

Accordingly, we hypothesized that genetic variants in E3 ubiquitin ligase-related genes may affect transcriptional regulation or ubiquitination efficiency, thereby altering protein turnover and signaling dynamics in ways that influence postoperative prognosis. We therefore investigated the association between potentially functional SNPs in E3 ubiquitin ligase-related genes and survival outcomes in patients with surgically resected NSCLC.

## 2. Materials and Methods

### 2.1. Study Population

This retrospective study included 744 patients with stage I–IIIA NSCLC who underwent surgical resection at Kyungpook National University Hospital (KNUH) between May 1999 and October 2020. The analysis of SNPs and clinical outcomes was conducted using a two-stage design. The 744 eligible patients were randomly divided into a discovery cohort (*n* = 380) and a validation cohort (*n* = 364) using a simple random allocation procedure without stratification. All participants were Korean, and written informed consent was obtained from each participant. The study protocol was approved by the Institutional Review Board of KNUH.

Genomic DNA used for genotyping was extracted from whole-blood samples collected before surgery. The blood samples were provided by the National Biobank of Korea at KNUH, which is supported by the Ministry of Health, Welfare and Family Affairs.

### 2.2. Selection of SNPs and Genotyping

A total of 65 candidate genes involved in E3 ubiquitin ligase-related processes were selected from the ubiquitin ligase reference tables provided by Cell Signaling Technology (https://www.cellsignal.com/learn-and-support/reference-tables/ubiquitin-ligase-table#anc59 (accessed on 20 September 2026)). Potentially functional SNPs were identified using the SNPinfo Web Server of the National Institute of Environmental Health Sciences (https://snpinfo.niehs.nih.gov/ (accessed on 20 September 2026)). Initially, 341 SNPs with a minor allele frequency of at least 0.10 in the HapMap Japanese in Tokyo population were identified. After linkage disequilibrium pruning, 160 SNPs remained. Of these, 65 SNPs predicted by FuncPred to have regulatory or functional effects were selected. One SNP could not be incorporated into the multiplex primer design and was therefore excluded. Among the 64 SNPs genotyped, 51 met the quality-control criteria of a call rate ≥ 95% and Hardy–Weinberg equilibrium *p* ≥ 0.05 and were included in the statistical analyses. Genotyping was performed using the MassARRAY iPLEX assay (SEQUENOM Inc., San Diego, CA, USA).

### 2.3. Cloning of BTRC Promoter Variants and Dual-Luciferase Reporter Assay

A dual-luciferase reporter assay was performed to determine whether rs10883617 T>C, located 887 bp upstream of the transcription start site, modulates the promoter activity of beta-transducin repeat-containing E3 ubiquitin protein ligase (BTRC). A 1085-bp fragment spanning positions −1029 to +56 and containing rs10883617 T>C was amplified from genomic DNA obtained from a heterozygous donor. The amplified fragment was cloned into the pGL3-Basic plasmid (Promega, Madison, WI, USA) using the NEBuilder HiFi DNA Assembly Master Mix Kit (New England Biolabs, Ipswich, MA, USA) in accordance with the manufacturer’s instructions. The primer sequences were as follows: BTRC_insert_fwd, 5′-tcgagatctgcgatctaagtGCATTTGGGTGTGTGTCAG-3′; BTRC_insert_rev, 5′-agtaccggaatgccaagcttTTGAGTGCCTTCTCTTGC-3′; pGL3-Basic_fwd, 5′-AAGCTTGGCATTCCGGTAC-3′; and pGL3-Basic_rev, 5′-ACTTAGATCGCAGATCTCG-3′. Overlapping sequences are indicated by lowercase letters. A549 and H1299 cells were obtained from the Korean Cell Line Bank (KCLB, Seoul, Republic of Korea). The cells were cotransfected with the pRL-SV40 vector (Promega, Madison, WI, USA) and the constructed pGL3-Basic plasmids using Effectene transfection reagent (QIAGEN, Hilden, Germany). After 48 h of incubation, the cells were harvested, and lysates were prepared using the Dual-Luciferase Reporter Assay System (Promega). Luciferase activity was measured using a Synergy HT Multi-Mode Microplate Reader (BioTek Instruments, Winooski, VT, USA). Firefly luciferase activity was normalized to Renilla luciferase activity to account for differences in transfection efficiency. All experiments were performed in triplicate.

### 2.4. RNA Preparation and Quantitative Reverse Transcription-PCR

BTRC mRNA expression was assessed using quantitative reverse transcription-PCR (qRT-PCR). Total RNA was isolated from tumor tissues and paired nonmalignant lung tissues from 167 patients using QIAzol Lysis Reagent (QIAGEN, Hilden, Germany). Real-time PCR was performed using a LightCycler 480 instrument (Roche Applied Science, Mannheim, Germany) and QuantiFast SYBR Green PCR Master Mix (QIAGEN, Hilden, Germany). The primer sequences were as follows: BTRC forward, 5′-CCCTTCTCGAACATACACCTAC-3′; BTRC reverse, 5′-TGTTGCTCATCCTGGTATTGG-3′; β-actin forward, 5′-TTGTTACAGGAAGTCCCTTGCC-3′; and β-actin reverse, 5′-ATGCTATCACCTCCCCTGTGT-3′. Each sample was analyzed in duplicate. Relative BTRC mRNA expression was normalized to β-actin expression and calculated using the 2^−ΔΔCt^ method.

### 2.5. Statistical Analysis

Differences in genotype distributions according to clinicopathologic characteristics were assessed using the χ^2^ test. Overall survival (OS) was defined as the interval from surgery to death or the date of last follow-up. Disease-free survival (DFS) was defined as the interval from surgery to recurrence or death from any cause. OS and DFS were estimated using the Kaplan–Meier method and compared using the log-rank test. Hazard ratios (HRs), 95% confidence intervals (CIs), and corresponding *p* values were estimated using multivariable Cox proportional hazards models adjusted for age, sex, smoking status, tumor histological type, pathological stage, and adjuvant therapy. Associations were evaluated under dominant, recessive, and codominant genetic models, and a *p* value < 0.05 was considered statistically significant. Differences in BTRC mRNA expression between paired tumor and nonmalignant lung tissues were evaluated using the Wilcoxon signed-rank test. Tumor BTRC mRNA expression was compared between patients with the TT genotype and carriers of the C allele (TC + CC) using the Mann–Whitney U test. Genetic association and survival analyses were performed using SAS for Windows, version 9.1 (SAS Institute, Cary, NC, USA). Statistical analyses of the qRT-PCR data and generation of the corresponding box plots were performed using R software, version 4.6 (R Foundation for Statistical Computing, Vienna, Austria) with the ggplot2 package, version 4.0.3.

## 3. Results

The clinicopathologic characteristics of the 380 patients in the discovery cohort and the 364 patients in the validation cohort are summarized in Tables S1 and S2, respectively. In the overall cohort of 744 patients, the 5-year OS and DFS rates were 74% and 55%, respectively (Table 1). Among the 51 potentially functional SNPs evaluated in the discovery cohort, 10 were significantly associated with OS or DFS (Table S3). Among these 10 SNPs, BTRC rs10883617 T>C was significantly associated with DFS in the validation cohort, whereas the association with OS was in the same favorable direction but did not reach statistical significance (Table S4). In the combined cohort, rs10883617 T>C was significantly associated with both OS and DFS. Carriers of at least one rs10883617 C allele, corresponding to the TC or CC genotype, had significantly better OS and DFS than patients with the TT genotype (HR = 0.55, 95% CI = 0.34–0.90, *p* = 0.02; and HR = 0.64, 95% CI = 0.46–0.89, *p* = 0.01, respectively; Table 2 and Figure 1).

The rs10883617 T>C polymorphism is located less than 1 kb upstream of BTRC and was predicted to influence promoter activity. To evaluate its functional effect, a luciferase reporter assay was performed to compare BTRC promoter activity between the two alleles. The rs10883617 C allele exhibited significantly lower promoter activity than the T allele in both A549 and H1299 lung cancer cell lines (both *p* < 0.01; Figure 2A). In addition, BTRC mRNA expression was significantly higher in tumor tissues from 167 patients with NSCLC than in paired nonmalignant lung tissues (*p* < 0.001; Figure 2B). However, BTRC mRNA expression did not differ according to rs10883617 genotype (Figure S1).

## 4. Discussion

This study evaluated the associations between potentially functional genetic variants in E3 ubiquitin ligase-related genes and clinical outcomes in patients with NSCLC who underwent curative-intent surgical resection. Among the 51 SNPs examined, BTRC rs10883617 T>C was significantly associated with DFS in the validation cohort, whereas the association with OS showed a concordant but statistically nonsignificant trend. In the combined cohort, carriers of the TC or CC genotype had significantly better OS and DFS than patients with the TT genotype. Moreover, luciferase reporter assays showed that the rs10883617 C allele conferred significantly lower BTRC promoter activity than the T allele in lung cancer cell lines, providing in vitro evidence of allele-specific regulatory function.

Tight control of protein homeostasis by the ubiquitin–proteasome system is essential for limiting oncogenic transformation and disease progression. Dysregulation of this system, particularly through altered stability of key regulatory proteins, has been associated with adverse clinical outcomes, including early tumor recurrence and reduced OS [10,11]. BTRC encodes β-TrCP1, the substrate-recognition component of the SCF^β-TrCP E3 ubiquitin ligase complex. Through recognition of phosphorylated degron motifs in target proteins, β-TrCP1 promotes their polyubiquitination and subsequent proteasomal degradation, thereby regulating critical cellular processes, including Wnt/β-catenin and NF-κB signaling, cell-cycle progression, and the DNA damage response [12,13,14]. Because β-TrCP1 targets both oncogenic and tumor-suppressive proteins, the biological consequences of aberrant BTRC expression or activity are likely to be highly context-dependent. Consistent with this complexity, alterations in BTRC expression or genomic status have been implicated in tumor development, metastatic progression, and clinical outcomes across multiple cancer types [15,16,17].

In the present study, BTRC mRNA expression was significantly higher in NSCLC tissues than in paired nonmalignant lung tissues, supporting a potential role for BTRC dysregulation in NSCLC biology. This result is consistent with a previous study demonstrating that β-TrCP1 promotes lung cancer cell proliferation and that elevated β-TrCP1 protein expression is associated with poorer OS in patients with lung cancer [18]. Collectively, these observations suggest that increased BTRC expression may contribute to tumor-promoting processes in at least a subset of NSCLC.

The rs10883617 T>C polymorphism is located within the proximal promoter region of BTRC, less than 1 kb upstream of the gene, and may therefore affect transcriptional regulation. Supporting this possibility, the rs10883617 C allele exhibited significantly lower promoter activity than the T allele in luciferase reporter assays. When considered alongside the favorable survival outcomes observed in the combined cohort among carriers of the C allele, these findings raise the possibility that allele-dependent variation in BTRC promoter activity alters β-TrCP1-mediated substrate turnover and thereby contributes to differences in NSCLC progression.

However, the molecular mechanisms underlying this association are likely complex. β-TrCP1 negatively regulates Wnt/β-catenin signaling by targeting phosphorylated β-catenin for ubiquitin-dependent proteasomal degradation, while promoting NF-κB activation through degradation of phosphorylated IκBα [13]. Consequently, reduced BTRC activity may exert divergent effects on these pathways rather than uniformly inhibiting or activating both. The favorable prognosis associated with the rs10883617 C allele may therefore reflect the net consequences of altered β-TrCP1-dependent degradation of multiple oncogenic and tumor-suppressive substrates rather than modulation of a single signaling pathway. The potential clinical relevance of these mechanisms is supported by previous studies linking the activation of Wnt and NF-κB signaling to postoperative recurrence and adverse outcomes in patients with early-stage NSCLC [19,20]. Nevertheless, neither pathway activity nor the abundance of individual β-TrCP1 substrates was directly evaluated in this study. Further assessment of β-TrCP1 protein expression, substrate abundance, and downstream signaling activity according to the rs10883617 genotype is needed to clarify the biological mechanism underlying the observed association.

Despite the allele-specific difference in reporter activity, BTRC mRNA expression did not differ significantly according to rs10883617 genotype in tumor tissues. This apparent discrepancy may reflect a modest or context-dependent regulatory effect, limited statistical power, tumor heterogeneity, or the influence of additional genetic and epigenetic factors on BTRC transcription. Moreover, reporter assays do not fully recapitulate the endogenous chromatin context. Accordingly, altered BTRC expression remains a biologically plausible but unconfirmed mediator of the association between rs10883617 and NSCLC prognosis.

BTRC rs10883617 was previously identified in a genome-wide association study as a candidate locus associated with poorer OS and possible gemcitabine resistance in advanced pancreatic cancer [21]. However, this association was not reproduced in an independent cohort [22]. These inconsistent findings suggest that the clinical effects of rs10883617 may vary according to cancer type, treatment setting, or study population. By contrast, the present study identified a prognostic association in surgically resected NSCLC and demonstrated allele-specific promoter activity, although direct comparisons with previous studies remain limited by differences in tumor biology, disease stage, and treatment exposure.

The principal strengths of this study include the relatively large cohort of 744 patients, the two-stage discovery and validation design, the consistent direction of the associations for both OS and DFS, and the integration of genetic association analyses with functional promoter assays and tumor-expression data. Several limitations should nevertheless be acknowledged. First, all participants were recruited at a single institution, which may limit the generalizability of the findings. Second, the allele-specific promoter activity observed in vitro was not accompanied by genotype-dependent differences in BTRC mRNA expression in clinical tumor specimens. Third, BTRC protein expression, β-TrCP1 substrate abundance, and downstream signaling activity were not assessed. Therefore, the clinical utility of rs10883617 remains to be established through independent multicenter validation, prospective studies, and dedicated analyses evaluating its potential role in risk stratification and prediction of treatment benefit. Since formal correction for multiple comparisons has not been performed, the risk of false positive results may be increased; therefore, results must be interpreted cautiously and verified in an independent cohort. In addition, molecular biomarker information, such as EGFR mutation status and PD-L1 expression, was not systematically available across the study period and therefore could not be incorporated into the prognostic analyses.

In summary, in the combined cohort of 744 patients with surgically resected NSCLC, carriage of the BTRC rs10883617 C allele was associated with significantly better OS and DFS. The C allele also exhibited lower BTRC promoter activity in lung cancer cell lines, supporting the potential functional relevance of this polymorphism. These findings indicate that BTRC rs10883617 may have value as a prognostic biomarker in surgically resected NSCLC; however, independent external validation and further mechanistic investigation are warranted.

## Figures and Tables

**Figure 1:**
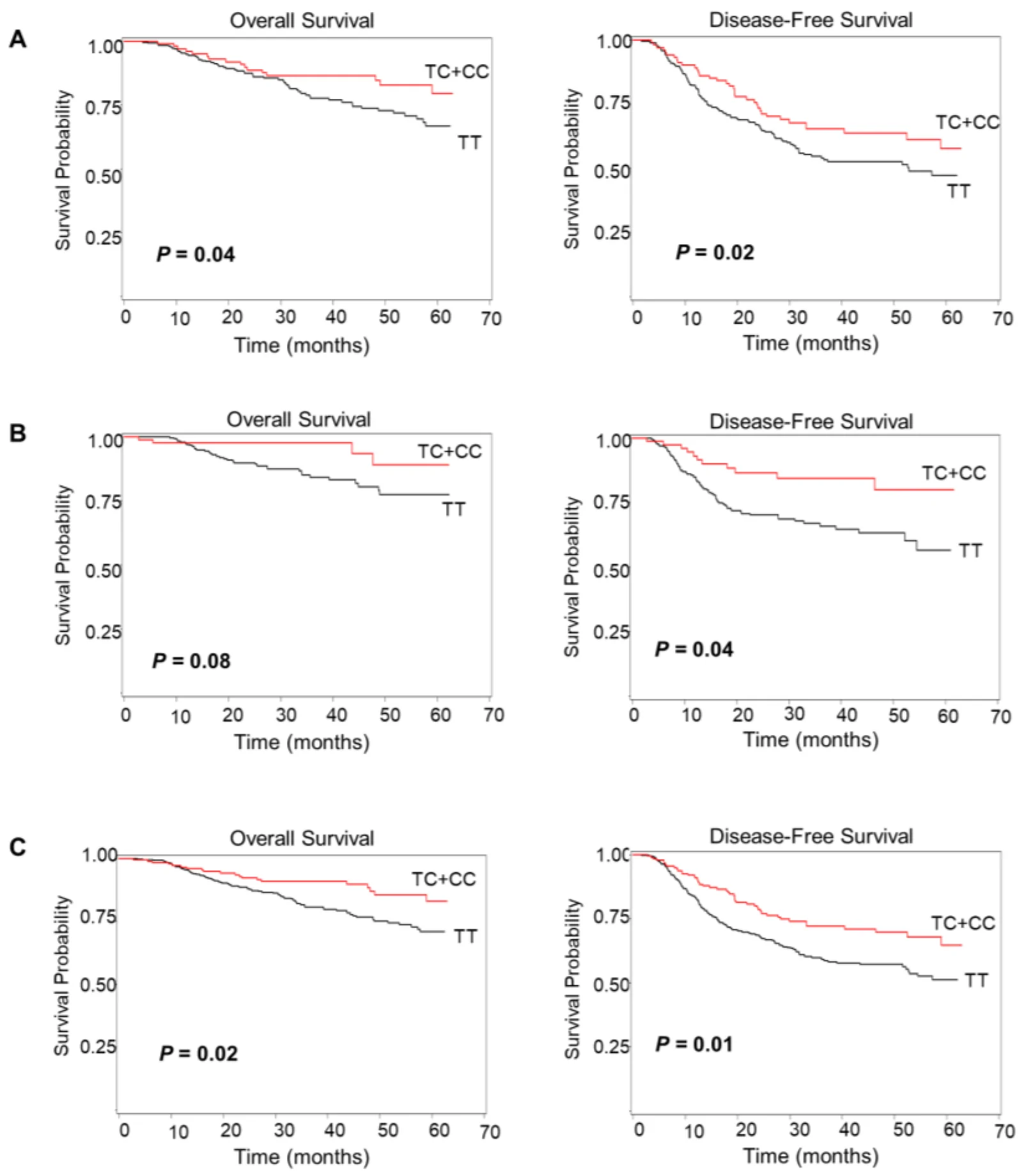
Overall survival and disease-free survival according to BTRC rs10883617 genotype. Kaplan–Meier curves for overall survival and disease-free survival in the discovery cohort (**A**), validation cohort (**B**), and combined cohort (**C**), stratified according to BTRC rs10883617 genotype under the dominant model. Adjusted *p* values were derived from multivariable Cox proportional hazards models including age, sex, smoking status, histological type, pathological stage, and adjuvant therapy as covariates.

**Figure 2:**
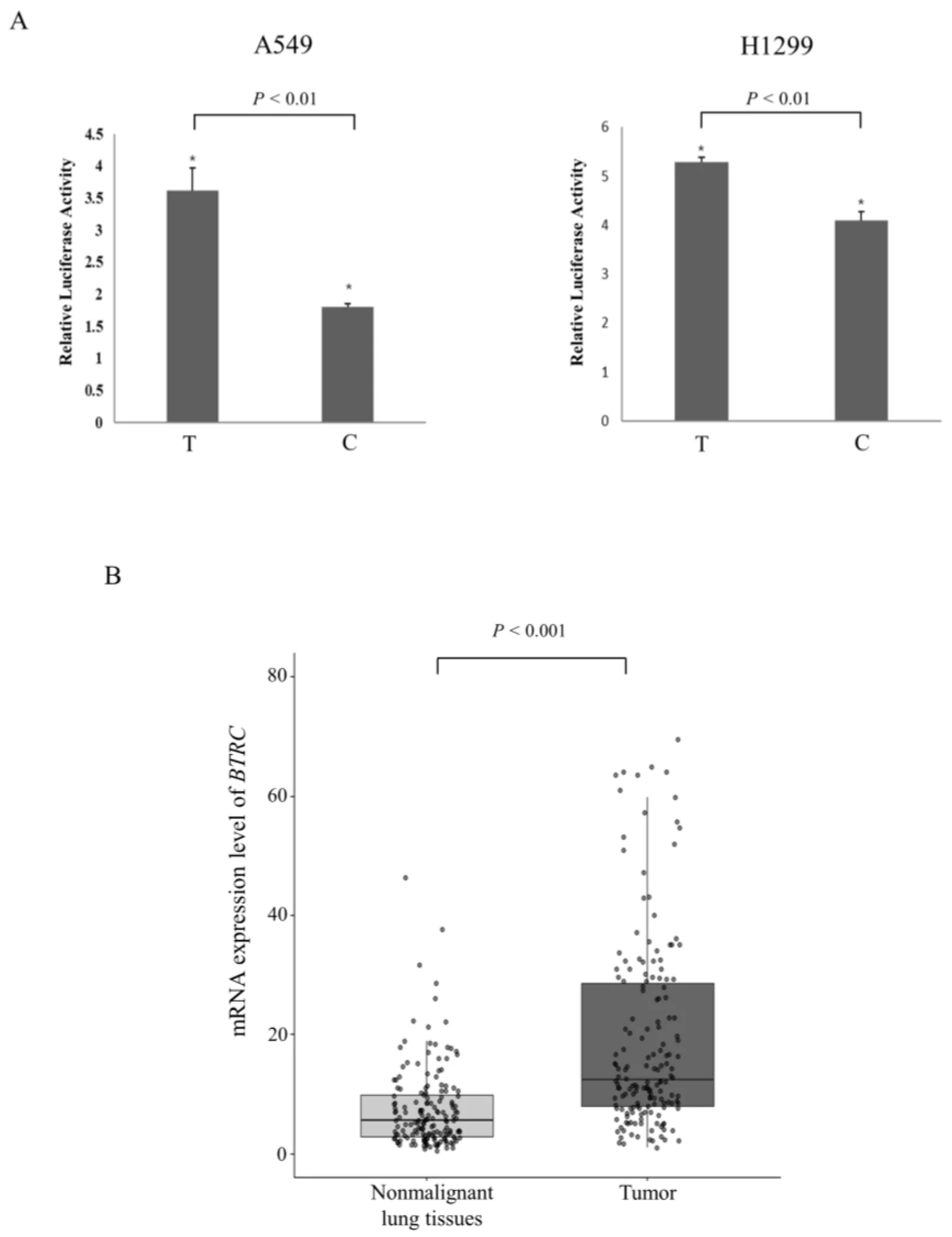
Allele-specific BTRC promoter activity and BTRC mRNA expression in non-small cell lung cancer. (**A**) Promoter activity of the rs10883617 T and C alleles in A549 and H1299 lung cancer cell lines, as determined using dual-luciferase reporter assays. (**B**) BTRC mRNA expression in 167 paired NSCLC tumor and nonmalignant lung tissues. Each scatter point represents the BTRC mRNA expression level in an individual tissue specimen.

**Table 1:** Clinicopathologic characteristics and univariate analyses of overall survival and disease-free survival in the entire study cohort (*n* = 744).

	Overall Survival				Disease-Free Survival		
Variables	No. of Cases	No. of Deaths (%) ^a^	5-Year OS (%) ^b^	Log-Rank *p*	No. of Events (%) ^a^	5-Year DFS (%) ^b^	Log-Rank *p*
Overal	744	129 (17.3)	74		248 (33.3)	55	
Age (years)							
<65	350	56 (16.0)	77	0.08	114 (32.6)	58	0.32
≥65	394	73 (18.5)	71		134 (34.0)	52	
Sex							
Female	267	25 (9.4)	85	<0.01	77 (28.8)	62	0.08
Male	477	104 (21.8)	68		171 (35.9)	51	
Smoking status							
Never	266	25 (9.6)	85	<0.01	77 (28.9)	60	0.10
Ever	471	104 (22.1)	67		169 (35.9)	52	
Pack-years ^c^							
<38	212	43 (20.3)	70	0.14	73 (34.4)	54	0.37
≥38	256	61 (23.8)	65		96 (37.5)	51	
Histological type							
SCC	237	59 (24.9)	64	<0.01	82 (34.6)	51	0.93
AC	493	66 (13.4)	79		161 (32.7)	57	
LCC	8	3 (37.5)	56		3 (37.5)	63	
NSCLC-NOS	6	1 (16.7)	80		2 (33.3)	60	
Pathologic stage							
I	495	60 (12.1)	80	<0.01	123 (24.9)	63	<0.01
II–IIIA	249	69 (27.7)	61		125 (50.2)	39	
Adjuvant therapy ^d^							
No	84	21 (25.0)	66	0.80	39 (46.4)	47	0.54
Yes	165	48 (29.1)	59		86 (52.1)	36	

Abbreviations: AC, adenocarcinoma; DFS, disease-free survival; LCC, large cell carcinoma; NSCLC-NOS, non-small cell lung cancer, not otherwise specified; OS, overall survival; SCC, squamous cell carcinoma. ^a^ Row percentage. ^b^ The 5-year overall survival and disease-free survival rates were estimated using the Kaplan–Meier method. ^c^ Evaluated among ever-smokers only. ^d^ Adjuvant therapy was evaluated among patients with pathological stage II–IIIA disease only.

**Table 2:** Associations between *BTRC* rs10883617 genotypes and overall survival and disease-free survival in the discovery, validation, and combined cohorts.

Cohort	Genotypes	Overall Survival					Disease-Free Survival			
		No. of Cases	No. of Deaths	5-Year OS	HR (95% CI) ^d^	*P* ^d^	No. of Events	5-Year-DFS	HR (95% CI) ^d^	*P* ^d^
		(%) ^a^	(%) ^b^	(%) ^c^			(%) ^b^	(%) ^c^		
Discovery ^e^	TT	259 (71.7)	63 (24.3)	67	1.00		112 (42.9)	47	1.00	
	TC	94 (26.0)	17 (18.1)	80	0.55 (0.30–0.99)	0.05	37 (35.1)	58	0.66 (0.44–0.98)	0.04
	CC	8 (2.2)	3 (37.5)	75	0.63 (0.15–2.69)	0.54	3 (37.5)	50	0.51 (0.16–1.64)	0.26
	Dominant	102 (28.2)	20 (19.6)	79	0.56 (0.32–0.98)	0.04	40 (39.2)	58	0.67 (0.48–0.95)	0.02
	Recessive	353 (97.8)	80 (22.7)	71	0.86 (0.42–1.77)	0.69	149 (42.2)	50	0.76 (0.43–1.36)	0.36
	Codominant				0.62 (0.38–1.02)	0.06			0.67 (0.48–0.95)	0.02
Validation ^e^	TT	269 (75.8)	35 (13.0)	77	1.00		76 (28.2)	57	1.00	
	TC	83 (23.4)	4 (4.8)	89	0.39 (0.13–1.12)	0.08	11 (13.2)	79	0.50 (0.26–0.98)	0.04
	CC	3 (0.8)	0 (0.0)	100	N.D.	0.99	0 (0.0)	100	N.D.	0.98
	Dominant	86 (24.2)	4 (4.8)	89	0.38 (0.13–1.11)	0.08	11 (12.8)	80	0.49 (0.25–0.96)	0.04
	Recessive	352 (99.2)	39 (11.5)	80	N.D.	0.99	87 (24.7)	62	N.D.	0.98
	Codominant				0.38 (0.13–1.10)	0.08			0.49 (0.25–0.94)	0.03
Combined	TT	528 (73.7)	98 (18.6)	71	1.00		188 (35.6)	51	1.00	
	TC	177 (24.7)	21 (11.9)	83	0.54 (0.32–0.90)	0.02	48 (27.1)	65	0.65 (0.47–0.91)	0.01
	CC	11 (1.5)	3 (27.7)	79	0.74 (0.18–3.04)	0.67	3 (27.3)	61	0.53 (0.17–1.68)	0.28
	Dominant	188 (26.3)	24 (12.8)	83	0.55 (0.34–0.90)	0.02	51 (27.1)	65	0.64 (0.46–0.89)	0.01
	Recessive	705 (98.5)	119 (16.9)	74	0.91 (0.45–1.85)	0.80	236 (33.5)	55	0.76 (0.43–1.36)	0.36
	Codominant				0.61 (0.39–0.95)	0.03			0.67 (0.50–0.90)	0.01

Abbreviations: CI, confidence interval; DFS, disease-free survival; HR, hazard ratio; N.D., not determined; OS, overall survival. ^a^ Column percentage ^b^ Row percentage. ^c^ The 5-year overall survival and disease-free survival rates were estimated using the Kaplan–Meier method. ^d^ HRs, 95% CIs, and corresponding *p* values were estimated using multivariable Cox proportional hazards models adjusted for age, sex, smoking status, histological type, pathological stage, and adjuvant therapy. ^e^ Genotyping failed for 19 patients in the discovery cohort and 9 patients in the validation cohort.

## Data Availability

Data are available for research purposes upon reasonable request to the corresponding author.

## Supplementary Materials

The following supporting information can be downloaded at: https://www.mdpi.com/article/10.3390/genes17101166/s1, Table S1: Clinicopathologic characteristics and univariate analyses of overall survival and disease-free survival in the discovery cohort (*n* = 380); Table S2: Clinicopathologic characteristics and univariate analyses of overall survival and disease-free survival in the validation cohort (*n* = 364); Table S3: List of analyzed 51 candidate single-nucleotide polymorphisms in the discovery cohort; Table S4: Validation analysis of the 10 SNPs associated with survival outcomes in the discovery cohort; Figure S1: BTRC mRNA expression according to rs10883617 genotype.
