# Supplementary material for: Association of BTRC rs10883617 with Survival Outcomes in Surgically Resected Non-Small Cell Lung Cancer
Source: Genes (Basel). 2026 Sep 22;17(10):1166. doi: 10.3390/genes17101166 (PMC13704296; doi:10.3390/genes17101166)
Supplement: Supplementary file 1 [file genes-17-01166-s001.zip › genes-4539628-supplementary.pdf]

Supplementary Materials

Table S1. Clinicopathologic characteristics and univariate analyses of overall survival and disease-free survival in the discovery cohort (n = 380).

Table S2. Clinicopathologic characteristics and univariate analyses of overall survival and disease-free survival in the validation cohort (n = 364).

Table S3. List of the 51 candidate single-nucleotide polymorphisms analyzed in the discovery cohort.

Table S4. Validation analysis of the 10 SNPs associated with survival outcomes in the discovery cohort.

Figure S1. BTRC mRNA expression according to rs10883617 genotype under the dominant genetic model.

Table S1. Clinicopathologic characteristics and univariate analyses of overall survival and disease-free survival in the discovery cohort (n = 380)

	Overall survival				Disease-free survival		
Variables	No. of cases	No. of death (%)[a]	5Y-OSR (%)[b]	Log-rank P	No. of death (%)[a]	5Y-DFSR (%)[b]	Log-rank P
Overall	380	87 (22.9)	71		159 (41.8)	51	
Age (years)							
<64	180	35 (19.4)	75	0.05	71 (39.4)	54	0.26
≥64	200	52 (26.0)	67		88 (44.0)	48	
Gender							
Female	136	17 (12.5)	84	< 0.01	49 (36.0)	58	0.14
Male	244	70 (28.7)	64		110 (45.1)	47	
Smoking status							
Never	135	18 (13.3)	84	< 0.01	51 (37.8)	57	0.25
Ever	241	69 (28.6)	64		107 (44.4)	47	
Pack-years[c]							
<38	107	34 (31.8)	61	0.91	50 (46.7)	45	0.81
≥38	133	35 (26.3)	66		57 (42.9)	49	
Histological type							
SCC	124	37 (29.8)	63	0.09	52 (41.9)	48	0.45
AC	250	48 (19.2)	76		104 (41.6)	53	
LCC	5	2 (40.0)	53		2 (40.0)	60	
NSCLC-NOS	1	0 (0.0)	100		1 (100.0)	0	
Pathologic stage							
I	254	43 (16.9)	76	< 0.01	84 (33.1)	59	< 0.01
II–IIIA	126	44 (34.9)	60		75 (59.5)	35	
Adjuvant therapy							
No	51	17 (33.3)	61	0.89	26 (51.0)	46	0.22
Yes	75	27 (36.0)	59		49 (65.3)	29	

Abbreviations: AC, adenocarcinoma; DFS, disease-free survival; LCC, large cell carcinoma; NSCLC-NOS, non-small cell lung cancer, not otherwise specified; OS, overall survival; SCC, squamous cell carcinoma.

[a]Row percentage.

[b]The 5-year overall survival and disease-free survival rates were estimated using the Kaplan–Meier method.

[c]Evaluated among ever-smokers only.

[d]In pathologic stage II–IIIA of cohort: 75 cases received adjuvant therapy.

Table S2. Clinicopathologic characteristics and univariate analyses of overall survival and disease-free survival in the validation cohort (n = 364)

Variables	Overall survival				Disease-free survival		
	No. of cases	No. of death (%)[a]	5Y-OSR (%)[b]	Log-rank *P*	No. of death (%)[a]	5Y-DFSR (%)[b]	Log-rank *P*
Overall	364	42 (11.5)	78		89 (24.5)	61	
Age (years)							
≤66	191	19 (10.0)	80	0.15	42 (21.9)	69	0.11
>66	173	23 (13.3)	75		47 (27.2)	52	
Gender							
Female	131	8 (6.1)	86	0.02	28 (21.4)	68	0.31
Male	233	34 (14.6)	74		61 (26.2)	58	
Smoking status							
Never	131	7 (5.3)	87	0.01	26 (19.9)	61	0.22
Ever	230	35 (15.2)	73		62 (27.0)	61	
Pack-years[c]							
<38	105	9 (8.6)	85	0.01	23 (21.9)	70	0.09
≥38	123	26 (21.1)	63		39 (31.7)	54	
Histological type							
SCC	113	22 (19.5)	68	0.01	30 (26.6)	57	0.92
AC	243	18 (7.4)	84		57 (23.5)	65	
LCC	3	1 (33.3)	67		1 (33.3)	67	
NSCLC-NOS	5	1 (20.0)	75		1 (20.0)	75	
Pathologic stage							
I	241	17 (7.1)	86	< 0.01	39 (16.2)	70	< 0.01
II–IIIA	123	25 (20.3)	62		50 (40.7)	45	
Adjuvant therapy							
No	33	4 (12.2)	81	0.34	13 (39.4)	54	0.78
Yes	90	21 (23.3)	59		37 (41.1)	43	

Abbreviations: AC, adenocarcinoma; DFS, disease-free survival; LCC, large cell carcinoma; NSCLC-NOS, non-small cell lung cancer, not otherwise specified; OS, overall survival; SCC, squamous cell carcinoma.

[a]Row percentage.

[b]The 5-year overall survival and disease-free survival rates were estimated using the Kaplan–Meier method.

[c]Evaluated among ever-smokers only.

[d]In pathologic stage II–IIIA of cohort: 90 cases received adjuvant therapy.

Table S3. List of analyzed 51 candidate single nucleotide polymorphisms in the discovery cohort.

							Overall survival						Disease-free survival					
							Dominant		Recessive		Codominant		Dominant		Recessive		Codominant	
SNP	Gene	1/1[a]	1/2[a]	2/2[a]	MAF	HWE *P*	*P*[b]	HR(95%CI)[b]	*P*[b]	HR(95%CI)[b]	*P*[b]	HR(95%CI)[b]	*P*[b]	HR(95%CI)[b]	*P*[b]	HR(95%CI)[b]	*P*[b]	HR(95%CI)[b]
rs1133505	MARCHF1	152	165	52	0.36	0.50	0.48	0.85(0.55-1.33)	0.43	0.76(0.39-1.50)	0.41	0.87(0.63-1.21)	0.91	0.98(0.71-1.36)	0.91	0.97(0.60-1.57)	0.89	0.98(0.78-1.24)
rs1351903	FBXW7	94	182	92	0.50	0.84	0.91	0.97(0.59-1.60)	0.39	0.80(0.48-1.33)	0.55	0.91(0.68-1.23)	0.16	0.77(0.54-1.11)	0.30	0.81(0.55-1.20)	0.13	0.84(0.67-1.06)
rs1660345	RNF19A	140	165	68	0.40	0.12	0.35	0.81(0.52-1.26)	0.47	1.23(0.71-2.11)	0.80	0.96(0.71-1.31)	0.54	0.90(0.65-1.25)	0.23	1.27(0.86-1.88)	0.82	1.03(0.82-1.28)
rs4140829	HECW1	261	100	15	0.17	0.17	0.08	1.49(0.95-2.32)	0.79	1.18(0.36-3.82)	0.11	1.35(0.93-1.95)	0.08	1.35(0.97-1.89)	0.14	1.69(0.84-3.36)	0.05	1.31(1.00-1.72)
rs4729514	SMURF1	209	132	32	0.26	0.09	0.33	1.24(0.80-1.92)	0.54	0.76(0.30-1.87)	0.59	1.10(0.78-1.53)	0.42	0.88(0.63-1.21)	0.21	0.65(0.33-1.27)	0.24	0.86(0.66-1.11)
rs10173538	MARCHF7	159	170	43	0.34	0.81	0.29	0.79(0.51-1.22)	0.59	1.19(0.63-2.25)	0.59	0.91(0.65-1.28)	0.55	0.90(0.65-1.26)	0.09	1.49(0.94-2.36)	0.71	1.05(0.82-1.35)
rs2269423	RNF5	155	161	54	0.36	0.25	0.18	1.37(0.87-2.18)	0.79	1.09(0.57-2.10)	0.27	1.20(0.87-1.66)	0.65	1.08(0.78-1.51)	0.20	0.71(0.41-1.20)	0.76	0.96(0.76-1.22)
rs3764990	MARCHF8	305	65	4	0.10	0.80	0.14	0.62(0.32-1.17)	0.11	3.36(0.77-14.6)	0.30	0.74(0.41-1.31)	0.71	0.92(0.60-1.41)	0.01	4.19(1.43-12.27)	0.82	1.05(0.71-1.54)
rs3821300	MARCHF7	179	156	41	0.32	0.43	0.19	1.35(0.86-2.12)	0.12	1.71(0.87-3.37)	0.08	1.34(0.96-1.88)	0.07	1.36(0.98-1.89)	0.13	1.50(0.88-2.55)	0.04	1.30(1.01-1.67)
rs5030416	TRAF6	263	95	16	0.17	0.06	0.31	1.28(0.80-2.04)	0.95	0.97(0.34-2.75)	0.46	1.15(0.79-1.66)	0.95	1.01(0.71-1.45)	0.27	0.62(0.27-1.45)	0.70	0.95(0.71-1.26)
rs702689	MAP3K1	237	119	21	0.21	0.24	0.31	0.79(0.49-1.25)	0.00	3.3(1.60-6.78)	0.76	1.06(0.73-1.55)	0.51	0.89(0.64-1.25)	0.00	2.52(1.46-4.37)	0.52	1.10(0.83-1.44)
rs10155	FZR1	282	81	7	0.13	0.68	0.80	0.93(0.56-1.57)	0.76	0.8(0.19-3.41)	0.74	0.93(0.60-1.45)	0.38	0.84(0.57-1.24)	0.37	0.59(0.18-1.88)	0.30	0.84(0.60-1.17)
rs1042852	CBLB	229	128	19	0.22	0.84	0.16	1.36(0.88-2.09)	0.11	1.99(0.85-4.66)	0.09	1.37(0.96-1.95)	0.20	1.23(0.90-1.70)	0.31	1.46(0.71-3.03)	0.15	1.22(0.93-1.60)
rs1046936	MIB1	163	147	51	0.34	0.06	0.15	1.41(0.88-2.26)	0.93	0.97(0.50-1.90)	0.32	1.17(0.86-1.60)	0.44	1.14(0.81-1.60)	0.86	1.04(0.65-1.66)	0.53	1.08(0.85-1.36)
rs11960484	MAP3K1	320	58	1	0.08	0.33	0.79	0.92(0.48-1.75)	0.99	N.D.	0.75	0.90(0.48-1.70)	0.22	0.74(0.45-1.20)	0.05	7.22(0.97-53.67)	0.31	0.78(0.49-1.26)
rs1642742	VHL	217	128	29	0.25	0.10	0.02	1.7(1.09-2.63)	0.41	1.37(0.65-2.90)	0.02	1.45(1.06-2.00)	0.66	1.08(0.78-1.49)	0.75	1.10(0.62-1.93)	0.64	1.06(0.83-1.35)
rs1859248	RFFL	272	89	12	0.15	0.17	0.38	1.23(0.77-1.97)	0.54	0.64(0.16-2.64)	0.56	1.12(0.76-1.66)	0.24	1.23(0.87-1.75)	0.98	1.01(0.41-2.50)	0.33	1.16(0.87-1.55)
rs2018246	SIAH2	130	177	69	0.42	0.52	0.34	0.81(0.52-1.26)	0.62	0.86(0.48-1.55)	0.37	0.87(0.64-1.18)	0.57	1.10(0.79-1.55)	0.94	0.98(0.65-1.50)	0.74	1.04(0.83-1.30)
rs2870820	MDM2	220	126	26	0.24	0.18	0.31	0.79(0.51-1.24)	0.58	1.25(0.56-2.77)	0.52	0.89(0.62-1.27)	0.99	1.00(0.72-1.39)	0.75	1.10(0.60-2.01)	0.90	1.02(0.79-1.32)
rs2901645	PELI1	294	64	3	0.10	0.81	0.85	0.94(0.52-1.70)	0.98	N.D.	0.60	0.87(0.50-1.49)	0.78	0.94(0.61-1.46)	0.98	N.D.	0.52	0.88(0.58-1.32)
rs7015490	WWP1	123	181	68	0.43	0.92	0.06	1.63(0.98-2.72)	0.85	1.06(0.59-1.88)	0.16	1.25(0.91-1.72)	0.95	1.01(0.71-1.44)	0.21	0.76(0.49-1.17)	0.50	0.92(0.73-1.16)
rs10883617	BTRC	259	94	8	0.15	0.88	0.04	0.56 (0.32-0.98)	0.69	0.86 (0.42-1.77)	0.06	0.62 (0.38-1.02)	0.02	0.67 (0.48-0.95)	0.36	0.76 (0.43-1.36)	0.02	0.67 (0.48-0.95)
rs12599315	STUB1	174	159	40	0.32	0.68	0.91	0.98(0.63-1.52)	0.73	1.14(0.54-2.39)	0.96	1.01(0.71-1.43)	0.29	1.19(0.86-1.65)	0.47	1.21(0.72-2.01)	0.25	1.15(0.90-1.47)
rs14062	MIB1	113	180	69	0.44	0.86	0.28	1.34(0.79-2.26)	0.63	0.86(0.46-1.61)	0.63	1.09(0.78-1.52)	0.97	0.99(0.69-1.42)	0.42	0.83(0.53-1.30)	0.63	0.94(0.74-1.20)
rs16844275	MARCHF7	287	82	1	0.11	0.05	0.77	0.93(0.55-1.55)	0.99	N.D.	0.81	0.94(0.56-1.57)	0.84	0.96(0.66-1.41)	N.D.	N.D.	0.84	0.96(0.66-1.41)

rs2293462	RNF34	306	66	3	0.10	0.79	0.36	1.3(0.74-2.29)	0.99	N.D.	0.41	1.26(0.73-2.17)	0.50	0.86(0.55-1.34)	0.98	N.D.	0.38	0.82(0.53-1.27)
rs2306541	CHFR	230	118	22	0.22	0.19	0.21	1.33(0.85-2.07)	0.25	1.58(0.73-3.45)	0.15	1.29(0.91-1.81)	0.90	1.02(0.73-1.43)	0.97	0.99(0.52-1.89)	0.93	1.01(0.78-1.32)
rs34862929	UBR5	312	47	2	0.07	0.87	0.45	0.75(0.36-1.57)	0.99	N.D.	0.45	0.76(0.37-1.56)	0.41	1.22(0.76-1.97)	0.98	N.D.	0.60	1.13(0.72-1.77)
rs6088512	ITCH	158	160	45	0.34	0.65	0.03	0.6(0.39-0.95)	0.85	0.93(0.46-1.88)	0.09	0.74(0.52-1.05)	0.09	0.75(0.54-1.04)	0.58	1.14(0.71-1.84)	0.32	0.88(0.69-1.13)
rs62323647	LNX1	190	154	29	0.28	0.77	0.04	1.59(1.03-2.46)	0.67	1.18(0.56-2.49)	0.08	1.33(0.97-1.82)	0.01	1.56(1.13-2.14)	0.74	1.10(0.62-1.98)	0.02	1.32(1.04-1.67)
rs6917033	UBR2	130	175	69	0.42	0.46	0.75	1.08(0.69-1.70)	0.45	1.24(0.71-2.15)	0.54	1.10(0.81-1.50)	0.64	1.08(0.77-1.52)	0.33	1.22(0.82-1.82)	0.40	1.10(0.88-1.38)
rs7248746	FZR1	295	73	5	0.11	0.84	0.59	0.86(0.49-1.50)	0.78	1.33(0.18-9.68)	0.66	0.89(0.53-1.50)	0.16	0.74(0.48-1.13)	0.84	0.86(0.21-3.53)	0.19	0.77(0.52-1.14)
rs7720490	MARCHF6	168	165	34	0.32	0.47	0.19	1.36(0.86-2.15)	0.59	0.81(0.36-1.79)	0.42	1.15(0.82-1.60)	0.80	0.96(0.69-1.33)	0.04	0.46(0.22-0.96)	0.25	0.86(0.67-1.11)
rs10417370	FZR1	295	73	5	0.11	0.84	0.80	0.93(0.54-1.61)	0.79	1.31(0.18-9.59)	0.86	0.96(0.57-1.59)	0.25	0.79(0.52-1.19)	0.83	0.85(0.21-3.50)	0.27	0.81(0.55-1.18)
rs11872553	MIB1	296	80	2	0.11	0.16	0.27	0.74(0.43-1.27)	0.98	N.D.	0.24	0.73(0.43-1.23)	0.15	0.74(0.49-1.12)	0.97	N.D.	0.11	0.72(0.49-1.07)
rs16260	CDH1	244	120	8	0.18	0.12	0.54	0.86(0.55-1.37)	0.73	0.70(0.10-5.13)	0.52	0.87(0.56-1.34)	0.02	0.65(0.45-0.92)	0.38	0.53(0.13-2.18)	0.02	0.66(0.48-0.93)
rs3796129	RNF168	265	101	10	0.16	0.92	0.45	1.19(0.75-1.90)	0.23	2.06(0.63-6.72)	0.32	1.23(0.82-1.85)	0.66	0.92(0.65-1.32)	0.07	2.17(0.94-5.01)	0.92	1.02(0.74-1.39)
rs4940386	NEDD4L	212	134	29	0.26	0.23	0.72	0.92(0.59-1.43)	0.34	1.45(0.68-3.10)	0.96	1.01(0.71-1.43)	0.86	1.03(0.75-1.42)	0.28	1.37(0.77-2.43)	0.56	1.08(0.84-1.39)
rs4985523	WWP2	147	175	49	0.37	0.79	0.78	0.94(0.60-1.47)	0.29	0.68(0.34-1.38)	0.47	0.89(0.64-1.23)	0.66	0.93(0.67-1.29)	0.67	0.90(0.55-1.46)	0.60	0.94(0.74-1.19)
rs6689407	TRIM63	213	140	24	0.25	0.88	0.93	1.02(0.66-1.58)	0.95	0.97(0.39-2.42)	1.00	1(0.70-1.43)	0.22	0.82(0.59-1.13)	0.05	1.78(0.99-3.19)	0.77	0.96(0.73-1.26)
rs1045979	CHFR	245	114	16	0.19	0.56	0.10	0.66(0.41-1.07)	0.78	0.85(0.26-2.76)	0.13	0.72(0.47-1.10)	0.72	1.07(0.76-1.50)	0.30	1.48(0.71-3.08)	0.50	1.1(0.83-1.47)
rs11119836	DTL	180	157	27	0.29	0.36	0.37	1.24(0.78-1.96)	0.60	1.23(0.56-2.71)	0.36	1.18(0.83-1.67)	0.77	0.95(0.68-1.33)	0.87	1.05(0.57-1.96)	0.87	0.98(0.75-1.28)
rs1196045	SYVN1	164	167	42	0.34	0.96	0.73	1.08(0.70-1.68)	0.84	1.08(0.53-2.21)	0.68	1.07(0.77-1.50)	0.98	1.00(0.73-1.39)	0.48	0.81(0.45-1.45)	0.76	0.96(0.75-1.23)
rs12376117	UHRF2	149	170	58	0.38	0.41	0.45	0.85(0.55-1.31)	0.51	0.81(0.43-1.51)	0.42	0.88(0.65-1.20)	0.50	0.89(0.64-1.24)	0.25	0.76(0.47-1.21)	0.28	0.88(0.70-1.11)
rs3820908	HECW2	287	78	9	0.13	0.19	0.70	0.9(0.53-1.53)	0.98	N.D.	0.34	0.80(0.50-1.28)	0.42	0.85(0.57-1.27)	0.56	0.70(0.22-2.27)	0.38	0.86(0.61-1.21)
rs57118874	SMURF2	179	163	26	0.29	0.17	0.73	0.92(0.59-1.45)	0.70	0.86(0.39-1.90)	0.70	0.93(0.66-1.32)	0.53	0.90(0.65-1.25)	0.77	0.91(0.49-1.72)	0.54	0.92(0.71-1.20)
rs7034754	RNF20	234	119	22	0.22	0.19	0.84	0.95(0.61-1.50)	0.67	0.80(0.29-2.22)	0.77	0.95(0.66-1.37)	0.69	1.07(0.77-1.49)	0.51	0.78(0.38-1.61)	0.96	1.01(0.77-1.31)
rs9837291	RNF168	164	167	41	0.33	0.88	0.13	1.42(0.90-2.24)	0.91	0.96(0.50-1.87)	0.30	1.18(0.86-1.61)	0.42	1.15(0.82-1.60)	0.60	0.87(0.53-1.45)	0.74	1.04(0.82-1.32)
rs2486143	HACE1	151	166	56	0.37	0.35	0.86	0.96(0.62-1.48)	0.09	1.57(0.93-2.65)	0.46	1.12(0.83-1.51)	0.11	0.77(0.56-1.07)	0.69	1.09(0.71-1.67)	0.38	0.90(0.72-1.13)
rs4648611	MIB2	242	119	17	0.20	0.63	0.75	1.08(0.68-1.70)	0.42	1.53(0.55-4.25)	0.56	1.12(0.76-1.66)	0.49	1.12(0.81-1.57)	0.82	1.10(0.51-2.36)	0.51	1.1(0.83-1.45)
rs7908745	MARCHF8	258	108	7	0.16	0.26	0.11	0.66(0.39-1.10)	0.59	0.58(0.08-4.20)	0.11	0.68(0.42-1.09)	0.55	0.90(0.63-1.28)	0.16	0.25(0.03-1.78)	0.32	0.85(0.61-1.18)

Abbreviation: CI, confidence interval; MAF, minor allele frequency; HR, hazard ratio; HWE, Hard-Weinberg equilibrium; N.D., not determined

[a]1 denotes a wild allele and 2 denote a polymorphic allele.

[b] HRs, 95% CIs, and corresponding P values were estimated using multivariable Cox proportional hazards models adjusted for age, sex, smoking status, histological type, pathological stage, and adjuvant therapy.

Table S4. Validation analysis of the 10 SNPs associated with survival outcomes in the discovery cohort

SNP	Gene	Allele	MAF	Overall survival						Disease-free survival					
				Dominant		Recessive		Codominant		Dominant		Recessive		Codominant	
				P[a]	HR(95%CI)[a]	*P*[a]	HR(95%CI)[a]	*P*[a]	HR(95%CI)[a]	*P*[a]	HR(95%CI)[a]	*P*[a]	HR(95%CI)[a]	*P*[a]	HR(95%CI)[a]
rs62323647	LNX1	TC	0.29	0.34	0.73(0.39-1.39)	0.39	0.53(0.13-2.23)	0.26	0.74(0.44-1.25)	0.08	0.68(0.43-1.05)	0.88	1.07(0.46-2.46)	0.18	0.77(0.53-1.12)
rs6088512	ITCH	GA	0.35	0.19	0.66(0.35-1.23)	0.74	1.20(0.40-3.59)	0.36	0.79(0.47-1.32)	0.49	0.86(0.56-1.32)	0.64	1.19(0.58-2.44)	0.74	0.94(0.67-1.33)
rs1642742	VHL	AG	0.23	0.14	0.59(0.30-1.19)	0.27	0.32(0.04-2.42)	0.10	0.61(0.34-1.1)	0.31	1.25(0.81-1.95)	0.40	0.60(0.19-1.95)	0.62	1.10(0.77-1.57)
rs702689	MAP3K1	GA	0.22	0.69	1.14(0.60-2.17)	0.67	0.65(0.09-4.77)	0.84	1.06(0.61-1.83)	0.96	1.01(0.66-1.56)	0.90	1.07(0.39-2.93)	0.93	1.02(0.70-1.47)
rs10883617	BTRC	TC	0.13	0.08	0.38(0.13-1.11)	0.99	N.D.	0.08	0.38(0.13-1.10)	0.04	0.49(0.25-0.96)	0.98	N.D.	0.03	0.49(0.25-0.94)
rs16260	CDH1	CA	0.18	0.49	0.78(0.38-1.59)	0.96	0.95(0.13-7.15)	0.53	0.82(0.43-1.55)	0.79	1.06(0.68-1.67)	0.84	0.87(0.21-3.6)	0.86	1.04(0.70-1.54)
rs3764990	MARCHF8	GA	0.09	0.35	0.64(0.24-1.66)	0.99	N.D.	0.30	0.62(0.25-1.54)	0.02	0.43(0.21-0.87)	0.98	N.D.	0.02	0.43(0.22-0.87)
rs7720490	MARCHF6	TG	0.75	0.43	0.77(0.40-1.47)	0.58	1.41(0.42-4.73)	0.66	0.88(0.51-1.53)	0.06	0.65(0.42-1.02)	0.37	0.59(0.18-1.89)	0.05	0.68(0.46-1.01)
rs3821300	MARCHF7	TC	0.32	0.37	1.36(0.70-2.65)	0.05	2.32(0.99-5.45)	0.11	1.48(0.91-2.39)	0.57	1.14(0.73-1.77)	0.35	1.37(0.72-2.61)	0.38	1.16(0.83-1.61)
rs4140829	HECW1	CG	0.17	0.29	0.67(0.32-1.42)	0.63	0.60(0.08-4.62)	0.30	0.71(0.37-1.35)	0.29	0.77(0.47-1.25)	0.24	0.30(0.04-2.23)	0.19	0.75(0.48-1.16)

Abbreviation: CI, confidence interval; MAF, minor allele frequency; HR, hazard ratio; N.D., not determined

[a]HRs, 95% CIs, and corresponding P values were estimated using multivariable Cox proportional hazards models adjusted for age, sex, smoking status, histological type, pathological stage, and adjuvant therapy.

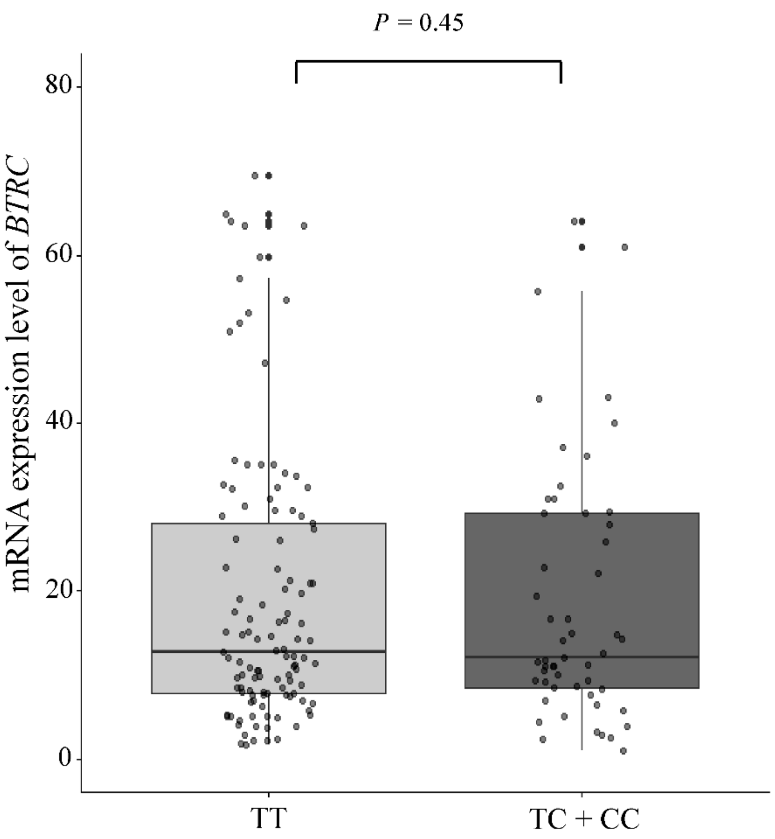

Figure S1. BTRC mRNA expression according to rs10883617 genotype under the dominant genetic model.
